# Methyl Potassium Siliconate and Siloxane Inhibit the Formation of Multispecies Biofilms on Ceramic Roof Tiles: Efficiency and Comparison of Two Common Water Repellents

**DOI:** 10.3390/microorganisms9020394

**Published:** 2021-02-15

**Authors:** Mattea Romani, Claire Carrion, Frédéric Fernandez, Philippe Lebaron, Raphaël Lami

**Affiliations:** 1 Laboratoire de Biodiversité et Biotechnologies Microbiennes, CNRS, Sorbonne Université, 66650 Banyuls-sur-Mer, France; romani@obs-banyuls.fr (M.R.); lebaron@obs-banyuls.fr (P.L.); 2 US042 INSERM—UMS 2015 CNRS (BISCEm), Université de Limoges, 87025 Limoges, France; claire.carrion@unilim.fr; 3 Microscopie Électronique Analytique (MEA), Université de Montpellier, 34095 Montpellier, France; frederic.fernandez@umontpellier.fr

**Keywords:** biodeterioration, ceramic roof tiles, multispecies biofilms, water-repellent, algal colonization, fungal colonization

## Abstract

Ceramic roof tiles are widespread marketed building materials, rapidly colonized by microorganisms that form multispecies biofilms on their surface and play crucial roles in biodeterioration processes. Coating tiles with water repellents is a pervasive industrial strategy employed to prevent liquid water penetration and slow biodeterioration. Very few studies have examined the links between the characteristics of water-repellent coatings and biofilm colonization patterns. Our work aims to compare the effects of coating tiles with two common water repellents (siliconate and siloxane) on the growth of colonizing microbes. We combined in situ exposure of tiles for over six years and macroscopic and microscopic observations with in vitro biotests, relying on the use of algal and fungal models. Our data showed that (1) tiles coated with water repellents were macroscopically less colonized by lichens (2) a significant fungal biofilm development at the microscopic scale (3) water repellents had very contrasting effects on our model strains. These data reinforce the great interest for industry to conduct more studies linking the nature of the water repellents with the composition of colonizing multispecies biofilms. The long-term objective is to improve the available water repellents and better adapt their selection to the nature of microbial colonization.

## 1. Introduction

Ceramic roof tiles are prevalent in outdoor building materials that are marketed worldwide [1]. Like any other building material, ceramic tiles deteriorate with time, which causes important economic losses [2,3]. This deterioration is induced not only by physical and chemical alterations of tiles but also by microbial biocolonization, which occurs soon after their manufacture and outdoor exposure [4]. Roof tiles are particularly prone to microbial colonization due to their high porosity. Indeed, the presence of pores promotes liquid water absorption, which is essential for microbial development [5,6] and favors the penetration of microorganisms into the material. Bacteria, algae, and fungi thus settle on tiles, forming multispecies biofilms embedded in a thick protective matrix that they synthesize [4,5,7,8,9,10]. Over time, these biofilms become mature and pigmented, producing black or green spots on roofs that are seen by customers as unaesthetic, and microorganism metabolic activities profoundly alter the composition of tiles. These biofilms also promote the settlement of macroorganisms such as plants and mosses [8,11,12] and ultimately cause significant alterations in tiles.

The use of water-repellent coatings is one of the most common antifouling strategies employed to limit the growth of biofilms on ceramic roof tiles. Water-repellent coatings have been developed since antiquity, where such uses of oil and wax have been reported [13]. In our modern times, hydrophobic compounds are commonly marketed to limit microbial settlement. The most commonly sold water repellents are silicon-based molecules [14]: Silanes, siloxanes, and siliconates are widely used for their antifouling capacities on building materials as they did not alter their appearance [14,15]. Water repellents increase the solid–liquid interfacial tension, resulting in apparent contact angles from 90° to 150° for droplets deposited on the material surface, and limit liquid water absorption [5]. These products present many advantages, such as UV resistance; adequate protection of natural building materials, such as granite; and a low production cost [14,16]. Due to their high alkali resistance, silanes are usually more frequently employed to protect concrete from waterproofing, as the compounds employed can deeply penetrate this type of material. Conversely, siliconates and siloxanes are usually more frequently employed to prevent ceramic materials from biofouling, thanks in particular to their water solubility and their high thermal stability, among other things [14,17].

Despite their large-scale adoption by industry and widespread usage in building construction, the impact of water repellents on microbial colonization has been examined in few studies. The effect of climate on building materials leads to the removal of the water-repellent coating over time, usually after 5 or 10 years of outdoor exposure [18]. Nevertheless, the microbial colonization of the coated tiles can start much earlier. For example, a previous study reported microbial colonization of mortars covered by water repellent after 15 months of outdoor exposure [15]. However, such studies remain very piecemeal, and whether the kinetics and patterns of microbial colonization are similar between ceramic materials covered by different types of water-repellent coatings remains largely unexplored.

Our study aims to characterize the efficiency of potassium methyl siliconate (PMS) and siloxane (Sx) in preventing multispecies biofilm development on ceramic roof tiles under natural exposure, combining in situ and in vitro experiments. We exposed ceramic roof tiles coated with these two distinct types of water-repellent compounds for over six years and then combined SEM, EDS analysis, and confocal macroscopy to characterize the extent of multispecies biofilm colonization. In addition, in vitro assays were carried out to evaluate the effectiveness of these two common water repellents in slowing down the growth of two eukaryotic ceramic roof tile colonizers: *Cladosporium cladosporioides*, a widespread melanized fungus [19,20], and *Stichococcus bacillaris,* a common lichenizing green alga [8,21,22].

Overall, our study aims to bridge the gap among many empirical observations conducted in industry and the need to accurately and scientifically evaluate the effects of water-repellent compounds to improve their efficiency. Thus, this work seeks to provide a framework to better evaluate the effects of marketed water repellents on the roof tile industry.

## 2. Materials and Methods

### 2.1. In Situ Exposure of Ceramic Roof Tiles and Sampling Strategy

Three distinct types of ceramic roof tiles were exposed in Aude (Occitanie, France), with a north/northwest orientation on the same types of displays, for 6 years to determine the long-term effects of tile waterproofing. All tiles were made with the same sand/clay mix at the same granulometry and fired at 980 °C. They were all red, plain, and without engobe. The first type of tile (UT) was tile untreated with water repellent and used as a control. The second type of tile (Sx) was tile treated with 2% siloxane commercial solution (Protectosil WS 610, Evonik, 1% of the active ingredient). The third type of tile (PMS) was tile treated with 1.7% of a commercial solution of potassium methyl siliconate (Silres BS16, Wacker, 0.6% of the active ingredient). The tiles have been treated according to the manufacturer’s instructions.

All tiles were collected after 6 years of exposure, visually examined, and photographed. Chlorophyll-*a* levels were measured by fluorimetry with a Benthotorch (Bbe, bbe Moldaenke GmbH, Schwentinental, Germany) fluorimeter, providing an estimation of the total photosynthetic cell (diatom, cyanobacteria, and green algae) count per mm^2^ (cells.mm^−2^) on each type of tile. All tiles were then fixed with paraformaldehyde (4% in PBS, Biotium, Fremont, CA, USA).

### 2.2. Evaluation of Hydrophobicity Features Induced by Water Repellents and after 6 Years of Exposure

The apparent contact angle on exposed tiles was measured after 6 years of exposure (protocol described below). In parallel, sterile tiles (similar to the UT type) were treated with increasing water-repellent concentrations in highly purified water. Tiles were coated with solutions at 0.1%, 0.5%, 1%, 2% and 5% Protectosil WS 610 or Silres BS16. To evaluate the hydrophobicity provided by the 2 tested water repellents at different concentrations and after 6 years of exposure, contact angles were measured (Table 1). On the tiles, 10 µL of methylene blue (1% solution, Sigma Aldrich, Saint-Louis, MO, USA) was deposited and imaged (Olympus Stylus, microscope mode with ISO 100). Images were analyzed with ImageJ (version: 2.0.0-rc-69/1.52p) and the DropSnake option to measure the apparent contact angles formed by the drop of methylene blue.

### 2.3. Microscopic Observations of In Situ Microbial Colonization

All tiles were observed by scanning electron microscopy (SEM), and confocal macroscopy following our routine and previously published protocols [4]. As negative controls, stained and unstained sterile tiles (autoclaved and calcined) were used.

#### Scanning Electron Microscopy and Energy Dispersive Spectrometry

Scanning electron microscopy (SEM) and energy dispersive spectrometry (EDS) analyses were carried out to observe colonization patterns of tile at a microscopic scale. Briefly, a small sample (2 cm × 2 cm) was observed using SEM (FEI Quanta FEG 200) with a 15 kV operational acceleration voltage at vacuum to 3.76e-1 Torr and a backscattered electron detector (BSED). An elementary analysis was performed using EDS. All observations were made at the University of Montpellier, MEA Platform.2.3.2. Fluorescence Macroscopy.

Tile samples were stained to evaluate and quantify microbial DNA and biofilm matrix using fluorescence macroscopy. Small samples (2 cm × 2 cm) were stained with SYTO 9 (10 µM, DNA intercalant, green, Invitrogen, Carlsbad, CA, USA) and FilmTracer SYPRO Ruby Biofilm Matrix Stain (ready to use protein labeling, red, Invitrogen, Carlsbad, CA, USA) [23].

Z stacking was obtained with a confocal macroscope (a Nikon AZ100 fluorescence macroscope with a CREST spinning disk head) using a 4× air objective and a 3× zoom and analyzed using Imaris software (version 9.6) and Biofilms Analysis XTension (Matthew J. Gastinger) to quantify the biovolume, the biomass, and the mean and max thicknesses. The biovolume is the sum of the volumes of all the objects found in the image and the biomass is the biovolume divided by the surface. Statistical differences between tiles were analyzed with the Wilcox test using the Ggpubr (0.4.0), Ggplot2 (3.3.2), and rstatix (0.6.0) R packages as implemented in R Studio (1.3.1056) with R 4.0.2 (2020-06-22) (Table 2) [24,25]. For the statistical analysis, 4 coupons and 5 images per coupons were used for NT tiles, and 2 coupons and 8 images per coupons were used for PMS and Sx tiles.

### 2.4. In Vitro Evaluation of the Water-Repellent Effects on Microbial Growth

Two eukaryotic models commonly found on ceramic roof tiles were used to evaluate the effects of increasing the concentrations of the tested water repellents: the fungal strain *Cladosporium cladosporioides* and the algal strain *Stichococcus bacillaris*. Both models were isolated from ceramic roof tiles by our team and previously described as tile colonizers [26,27,28,29].

#### 2.4.1. Evaluation of Water-Repellent Effects on Fungal Growth

The effects of the water repellents were first tested on *Cladosporium cladosporioides* using two different sets of culture conditions: (i) a minimum medium, MS (according to MIL-STD-810 Environmental Engineering Considerations and Laboratory Tests) with 15 g·L^−1^ agar (Sigma Aldrich, Saint-Louis, MO, USA), and (ii) a rich medium, potato dextrose agar (PDA, Sigma Aldrich). In these media, increasing concentrations (0.1%, 0.5%, 1%, 2% and 5%) of Protectosil WS 610 and Silres BS16 were added, and the solution pH was measured with a pH meter (Accumet AE150, Fischer Scientific, Waltham, MA, USA) (Table 3). Then, 6-well plates were prepared with each solution in triplicate, and no addition of water repellents was used as a control.

A preculture of *Cladosporium cladosporioides* was achieved on PDA, and then a spore suspension was prepared according to the norm NF EN ISO 846:2019-04 (Plastics. Evaluation of the action of microorganisms). Briefly, the Petri dish was scraped, and fungal mycelium was deposited in a sterile tube containing 5 mL of MS. Sterile glass beads were added, and the tube was vortexed for 3 min. Then, the suspension was filtered through a 20 µm filter, sterile glass beads were added again, and the tube was vortexed for 1 min. Finally, the suspension was diluted in 50 mL of MS, and spores were counted under an optical microscope with a Malassez cell (depth 0.200 mm, Marienfeld, Germany). A total of 2.5 × 10^4^ cells were inoculated in each well of the plates. The fungal growth was then monitored over 28 days. The growth diameters were measured after 3, 5, 7, 14, 21, and 28 days on PDA and after 7, 14, 21, and 28 days on MS. Statistical differences between the samples with different concentrations of water repellents and the control were calculated with a *t*-test corrected with the Benjamini–Hochberg procedure (FDR) using Ggpubr (0.4.0), Ggplot2 (3.3.2), and rstatix (0.6.0) R packages as implemented in R Studio (1.3.1056) with R 4.0.2 (2020-06-22) and [24,25]. Results significance: ns: non-significative *p* value > 0.5, * 0.05 ≥ *p* value > 0.01, ** 0.01 ≥ *p* value > 0.001, *** 0.001 ≥ *p* value > 0.0001, **** 0.0001 ≥ *p* value.

#### 2.4.2. Evaluation of Water-Repellent Effects on Algal Growth

Following a similar approach as for *Cladosporium cladosporioides*, the impact of water repellents on *Stichococcus bacillaris* was evaluated. Briefly, 6-well plates were prepared with 0%, 0.1%, 0.5%, 1%, 2% and 5% of both water repellents diluted in BG11 (BG11 50X with 15 g·L^−1^ agar, Sigma Aldrich) [30,31]. *Stichococcus bacillaris* cells (2.5 × 10^4^*)* were also inoculated in triplicate for each concentration of water repellents. Algal growth was followed for 28 days, and growth diameters were measured after 7, 14, 21, and 28 days. Statistical differences were also calculated using a similar test as described above for *Cladosporium cladosporioides*.

## 3. Results

### 3.1. Macroscopical Aspects of Ceramic Roof Tiles after 6 Years of Exposure with or without an Initial Water-Repellent Coating.

#### 3.1.1. Macroscopic Observations

After 6 years of exposure, all tiles were collected, and the apparent contact angles were measured. No tile presented a hydrophobic feature (contact angle = 0° for all samples), even those initially coated with a water repellent. This observation revealed that all the water-repellent coatings were leached after 6 years of exposure, which was reinforced by the values of the contact angles on tiles coated at different concentrations of PMS or Sx (Table 1).

**Figure 1:**
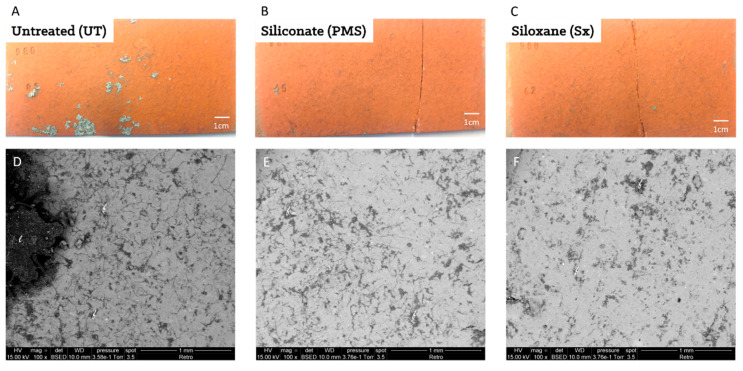
Macroscopic and backscattered SEM images of the biofilm colonizing tiles exposed 6 years: (**A**–**C**) 1 cm scale: (**A**) UT tile, (**B**) PMS tile, and (**C**) Sx tile. (**D**–**F**) 1 mm scale: (**D**) UT tile, (**E**) PMS tile, and (**F**) Sx tile. In the pictures, fungal filaments are represented by the letter f, and pioneer lichens by l.

Then, the colonization of tiles was observed and photographed (Figure 1A–C): only the initially noncoated tiles were colonized by gray leafy lichens, presenting the typical morphological characteristics of *Physcia* species (Figure 1A). However, all the tiles, even the initially coated tiles, presented typical spots due to microbial colonization by melanized fungi (Figure 1A–C). No chlorophyll-*a* fluorescence was detected on any tile, confirming the observation that tiles were mainly colonized by fungi and probably in a much lower proportion by microscopic algae.

#### 3.1.2. SEM Observations and EDS Analysis

Backscattered images confirmed that all the tile types were colonized (Figure 1D–F). The initially uncoated tiles clearly were the tiles most colonized with lichens and extensive filamentous fungal development (Figure 1D). On the two initially coated tiles (with Sx and PMS), less but important fungal colonization was visible as long and typical filaments. The organic nature of these microscopic structures was confirmed by EDS analysis, which revealed that the presence of carbon correlated with these filamentous structures (Table S1).

#### 3.1.3. Confocal Macroscopy Observations

**Figure 2:**
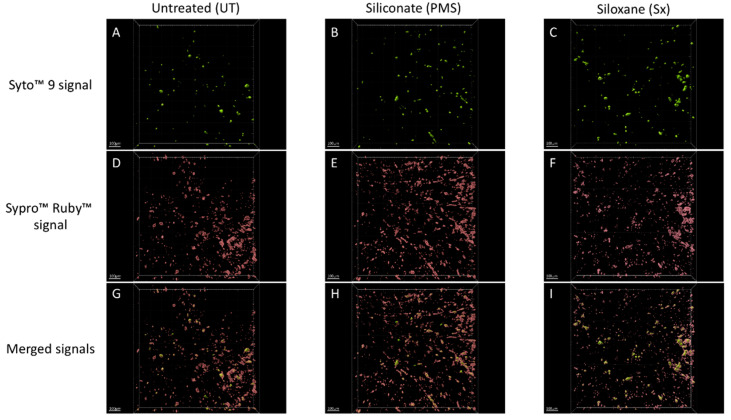
Fluorescent macroscopic images of the biofilm colonizing tiles exposed 6 years (100µm scale): (**A**–**C**) Syto 9 signal: (**A**) UT tile, (**B**) PMS tile, and (**C**) Sx tile. (**D**–**F**) Sypro Ruby signal: (**D**) UT tile, (**E**) PMS tile, and (**F**) Sx tile. (**G**–**I**) Syto 9 and Sypro Ruby merged signals: (**G**) UT tile, (**H**) PMS tile, and (**I**) Sx tile.

Confocal macroscopy observations revealed the extent of microbial colonization (microbe DNA was strained using SYTO™ 9) and underlined the importance of the biofilm matrix (proteins of the matrix were stained using FilmTracer SYPRO Ruby) (Figure 2). Interestingly, the distribution of cells on the tile surfaces and the distribution of the biofilm matrix appeared relatively homogeneous.

Thus, confocal macroscopy observations provided interesting data that characterized the multispecies biofilm matrix features. On all types of tiles, this matrix appeared thick and well-developed. The average biovolumes of the matrices were 1851.5 ± 916.8 µm^3^ for UT, 1624.8 ± 546.1 µm^3^ for PMS, and 5280.8 ± 8979.3 µm^3^ for Sx. The biomasses of the matrices were, on average, 443.2 ± 219.5 µm^3^·µm^−2^ for UT, 389.0 ± 130.7 µm^3^·µm^−2^ for PMS, and 1264.1 ± 2150.0 µm^3^·µm^−2^ for Sx. The mean thicknesses of the biofilms were 26.1 ± 7.0 µm for UT, 22.0 ± 1.4 µm for PMS, and 24.7 ± 4.0 µm for Sx. The maximal thicknesses of the biofilms were 91.8 ± 31.8 µm for UT, 103.5 ± 20.1 µm for PMS, and 111.6 ± 23.5 µm for Sx (Table 2). Nevertheless, no significant difference among matrix thicknesses was found by comparing the different types of tiles (Wilcox test, *p*-value > 0.5, Table 2).

### 3.2. In Vitro Assays

#### 3.2.1. Evaluation of Water-Repellent Effects on the Growth of the Fungal Model *Cladosporium cladosporioides* on a Minimal Medium Untreated or Amended with Silane or Siloxane

**Figure 3:**
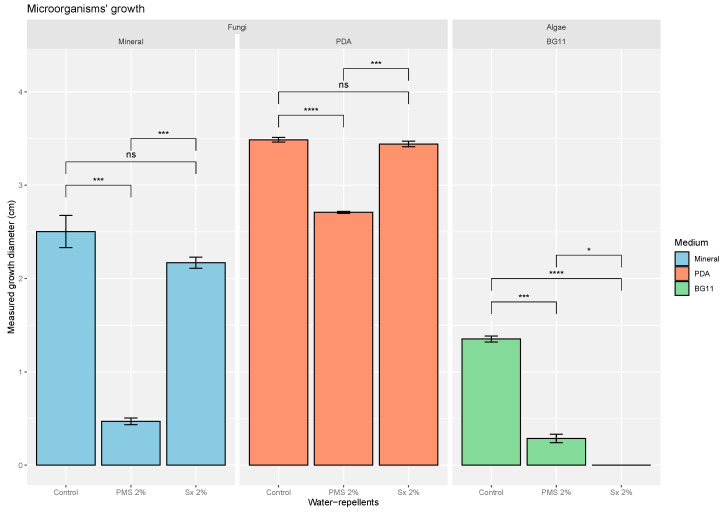
Fungal growth (*Cladosporium cladosporioides*) on mineral medium and PDA after 28 days with commercially used water-repellent concentrations (PMS 2%, and Sx 2%) and without water-repellent addition as the negative control. Algal (*Stichococcus bacillaris)* growth on BG11 after 28 days with commercially used concentrations of water repellents (PMS 2%, and Sx 2%) and without water-repellent addition as the negative control. ns: non-significative, * 0.05 ≥ *p* value > 0.01, ** 0.01 ≥ *p* value > 0.001, *** 0.001 ≥ *p* value > 0.0001, **** 0.0001 ≥ *p* value.

We found significant growth of our fungal model *Cladosporium cladosporioides* on minimal medium, with an average growth diameter of 2.50 ± 0.42 cm for the fungal biofilm after 28 days (Figure 3). Additionally, significant growth in the minimal medium amended with 2% PMS was measured (0.47 ± 0.06 cm), which was significantly less than that of the control (−81.2%; *t*-test, *p*-value 1.16 × 10^−4^ ***) after 28 days. Growth in the presence of siloxane (2%) was also measured (2.17 ± 0.10 cm), but the growth was not significantly different from that of the control (*t*-test, *p*-value 1.33 × 10^−1^, ns).

In addition, the influences of PMS and Sx concentrations (from 0 to 5%) were determined by measurements at 7, 14, 21, and 28 days (Figure S1). This detailed analysis of fungal growth confirmed the main trends described previously: both growths were affected significantly in most of the test cases by the water-repellent concentrations, and no growth was observed in the presence of 5% PMS. It is worth noting that the pH was high in cultures grown in the presence of PMS (9.75–12.18) (Table 3).

#### 3.2.2. Evaluation of Water-Repellent Effects on the Growth of the Fungal Model *Cladosporium cladosporioides* on a Rich Medium (PDA)

We found significant growth of the model fungus *Cladosporium cladosporioides* on PDA medium, with an average fungal biofilm growth diameter of 3.49 ± 0.06 cm observed after 28 days (Figure 3). Additionally, growth on PDA with 2% PMS (2.70 ± 0.02 cm) was significantly less than that of the control (−22.3%; *t*-test, *p*-value 4.73 × 10^−7^ ***) after 28 days. Important growth in the presence of siloxane (2%) was also measured (3.44 ± 0.05 cm), but the value was not significant compared to that of the control (*t*-test, *p*-value 3.06 × 10^−1^ ns).

Moreover, the influences of PMS and Sx concentrations (from 0 to 5%) were determined by measurements at 3, 5, 7, 14, 21, and 28 days (Figure S2). This detailed analysis of fungal growth confirmed the main trends described previously; both growths were clearly affected significantly in most test cases by the water-repellent concentrations (especially for PMS), and no growth was observed at 5% PMS. It is worth noting that the pH was high in cultures grown in the presence of PMS (10.05–12.05) (Table 3).

#### 3.2.3. Evaluation of Water-Repellent Effects on the Growth of the Algal Model *Stichococcus bacillaris* on the Minimal Medium BG11

We found significant growth of the model alga *Stichococcus bacillaris* on BG11, a medium adapted to algal growth, with an average growth diameter of 1.35 ± 0.08 cm for the algal biofilm after 28 days (Figure 3). Additionally, growth in BG11 with 2% PMS (0.29 ± 0.08 cm) was significantly less than that of the control (−78,5%; *t*-test, *p*-value 1.03 × 10^−4^ ***) after 28 days. Finally, no growth was measured in BG11 with 2% Sx, whose value was significantly lower than those of the control and the 2% PMS case (*t*-tests, *p*-values 6.30 × 10^−7^ **** and 3.09 × 10^−2^ *, respectively).

In addition, the influences of PMS and Sx concentrations (from 0 to 5%) were determined by measurements at 7, 14, 21, and 28 days (Figure S3). This detailed analysis of algal growth confirmed the main trends described previously: Both growths were clearly affected significantly in most of the test cases by the water-repellent concentrations, and no growth was observed at 5% PMS and 1, 2, and 5% Sx. Again, it is worth noting that the pH was high in cultures grown in the presence of PMS (10.90–12.43) (Table 3).

## 4. Discussion

The use of water repellents as an antifouling strategy to limit the growth of multispecies biofilms on ceramic building materials is widespread and appreciated by both manufacturers and building owners [1]. Many ceramic roof tiles are thus coated during their manufacturing or after roof cleaning ordered by customers to prevent microbial settlement by reducing water uptake [14,17]. Water-based silicones offer excellent cost efficiency [17,32,33]. Nevertheless, the extensive and widespread use of water repellents largely relies on empirical observations, and very few scientific studies have focused on microbial colonization kinetics and the importance of such coatings [15]. Such studies would be of great interest for the tile industry, as they could significantly improve the way these commonly marketed antifouling solutions are used.

Our study evaluated the growth of multispecies biofilms on ceramic roof tiles coated with water-repellent coatings composed of potassium methyl siliconate or siloxane. After six years of exposure under a Mediterranean climate, both water repellents were completely leached out from tiles, as shown by the apparent contact angles that we measured. Nevertheless, at the macroscopic level, significant differences were observed, as tiles that were initially uncoated were colonized by lichens more than the initially coated tiles. Thus, both water repellents clearly delayed colonization, especially that by lichens.

However, our SEM and confocal macroscopy observations revealed important colonization of all tile types by a fungal biofilm. Additionally, the biofilm matrix was thick on both initially coated and uncoated tile types. This important colonization is not surprising regarding results in the literature. For example, fungal colonization of water-repellent-coated mortars was detected after 15 months of exposure [15]. Conversely, mixing water repellents as polysiloxane with biocidal molecules such as copper nanoparticles has given interesting results by lowering the biological colonization of stones after 30 months of outdoor exposure [34]. However, such studies remain scarce, and one original aspect of our work is that it provides new data based on confocal macroscopy observations. Indeed, our fluorescence-based macroscopy observations revealed the presence of a thick and significant biofilm matrix that embedded cell clusters similarly among the three types of studied tiles. Thus, a microbial biofilm clearly and significantly colonized the tiles before the appearance of the macroscopically visible lichens. We did not detect microscopic algae colonizing the tiles. Such observations are probably linked with the Mediterranean climate under which tiles were exposed, as wetter climates favor microalgal colonization [35,36,37].

Our in vitro assays provided interesting clues to better understand the microbial patterns of roof-tile colonization and revealed major differential effects of the water repellents on the colonizing fungi and algae (Supplementary Figures S1–S3). First, more than 1% siloxane and 5% PMS completely inhibited *Stichococcus bacillaris* development, while the effects on *Cladosporium cladosporioides* were much less marked. On the other hand, PMS deeply impacted both our algal and fungal strains but did not fully inhibit their growth. The effect of PMS is probably based on the increase in pH after its addition, as already mentioned in previous studies [38]. Nevertheless, and very clearly, our data acquired in vitro revealed the need to adequately select a water-repellent coating by considering the type of microbial colonization that occurs in the area where the tiles are exposed. For example, it has already been shown that algae dominate on wet and shaded surfaces, and conversely, fungi are more prevalent in areas with more sun exposure [39]. Therefore, the correct choice of water repellent that affects one or the other type of microorganism is essential to prevent multispecies biofilm colonization over a long period.

Overall, our study provided data to better explain the empirical observations of the effectiveness of water repellents against microbial colonization. Thus, our study highlighted the need to better evaluate the effects of the large diversity of marketed water-repellent coatings on the microbial diversity growing on tiles and other types of building materials. If we provided here data related to the effects of water repellents on fungal and microalgal diversity, an additional step to obtain deeper observations would undoubtedly be to take advantage of recent high-throughput DNA sequencing technologies that provide a detailed picture of microbial diversity. Moreover, it could be interesting to evaluate the efficiency of water repellents in vitro on multispecies biofilms. However, algal–fungal co-cultures remain challenging and species-dependent [40]. Our study also revealed the value of selecting the type of water-repellent treatment on a case-by-case basis considering the environment in which the tiles are exposed to properly sell the most suitable coatings and optimize their effectiveness over time. This report also reinforces the need to strengthen the links between the ceramic roof-tile industry and environmental microbiologists to meet this challenge.

## Figures and Tables

**Table 1:** Apparent contact angles measured on tiles after water-repellent coatings of siliconate (PMS) and siloxane (Sx) (at 0, 0.1, 0.5, 1, 2, and 5%) and after 6 years of exposure.

Water-Repellent	Concentration (% Commercial Solution)	Contact Angle (°)
No	0%	0
PMS	0.1%	79
	0.5%	95
	1%	95
	2%	97
	5%	106
Sx	0.1%	100
	0.5%	104
	1%	104
	2%	104
	5%	116
No—6 years	0%	0
PMS—6 years	1.7%	0
Sx—6 years	2%	0

**Table 2:** Macroscopy analysis of biomass (µm^3^·µm^−2^), biovolume (µm^3^), mean and mean thickness (µm) with statistical analysis (Wilcox test), ns not significant.

		Biomass	Biovolume	Mean Thickness	Max Thickness
Tile	UT	443.2 ± 219.5	1851.5 ± 916.8	26.1 ± 7.0	91.8 ± 31.8
	PMS	389.0 ± 130.7	1624.8 ± 546.1	22.0 ± 1.4	103.5 ± 20.1
	Sx	1264.1 ± 2150.0	5280.8 ± 8979.3	24.7 ± 4.0	111.6 ± 23.5
*p* value	UT/PMS	0.704 ns	0.704 ns	0.099 ns	0.347 ns
	UT/Sx	0.704 ns	0.704 ns	0.765 ns	0.11 ns
	MPS/Sx	0.552 ns	0.552 ns	0.238 ns	0.334 ns

**Table 3:** pH measures of solutions containing Sx and PMS at 0, 0.1, 0.5, 1, 2 and 5%.

Medium	Water-Repellent	Concentration (% Commercial Solution)	pH Solution
MS	No	0%	6.41
	PMS	0.1%	9.75
		0.5%	10.91
		1%	11.37
		2%	11.72
		5%	12.18
	Sx	0.1%	6.30
		0.5%	6.24
		1%	6.20
		2%	6.18
		5%	6.31
PDB	No	0%	5.00
	PMS	0.1%	10.05
		0.5%	10.95
		1%	11.36
		2%	11.57
		5%	12.05
	Sx	0.1%	5.88
		0.5%	5.25
		1%	5.30
		2%	5.30
		5%	5.60
BG11	No	0%	6.81
	PMS	0.1%	10.90
		0.5%	11.48
		1%	11.72
		2%	11.99
		5%	12.43
	Sx	0.1%	6.99
		0.5%	7.26
		1%	7.32
		2%	7.11
		5%	8.27

## Data Availability

All raw data are available in the paper and the supplementary material.

## Supplementary Materials

The following are available online at https://www.mdpi.com/2076-2607/9/2/394/s1, Supplementary Figure S1: Monitoring of fungal growth (*Cladosporium cladosporioides*) on mineral medium after 7, 14, 21, and 28 days with increasing concentrations of PMS and Sx (0, 0.1, 0.5, 1, 2, and 5%). Supplementary Figure S2: Monitoring of fungal growth (*Cladosporium cladosporioides*) on PDA after 3, 5, 7, 14, 21, and 28 days with increasing concentrations of PMS and Sx (0, 0.1, 0.5, 1, 2, and 5%). Supplementary Figure S3: Monitoring of algal growth (*Stichococcus bacillaris*) on BG11 after 7, 14, 21, and 28 days with increasing concentrations of PMS and Sx (0, 0.1, 0.5, 1, 2, and 5%). Supplementary Table S1: EDS analysis of relevant spectra on UT, PMS, and Sx tiles after 6 years of exposure in Occitanie (atomic%).
