# Supplementary material for: Methyl Potassium Siliconate and Siloxane Inhibit the Formation of Multispecies Biofilms on Ceramic Roof Tiles: Efficiency and Comparison of Two Common Water Repellents
Source: Microorganisms. 2021 Feb 15;9(2):394. doi: 10.3390/microorganisms9020394 (PMC7918968; doi:10.3390/microorganisms9020394)
Supplement: Supplementary file 1 [file microorganisms-09-00394-s001.pdf]

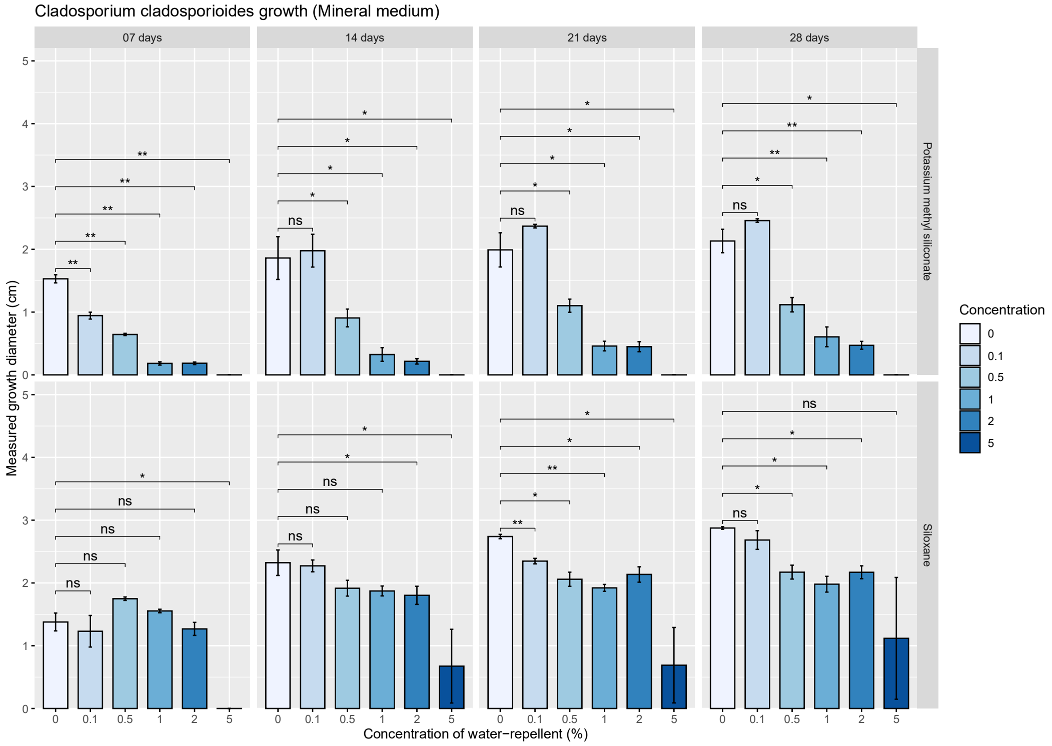

Figure S1. Monitoring of fungal growth (*Cladosporium cladosporioides*) on mineral medium after 7, 14, 21, and 28 days with increasing concentrations of PMS and Sx (0, 0.1, 0.5, 1, 2, and 5%).

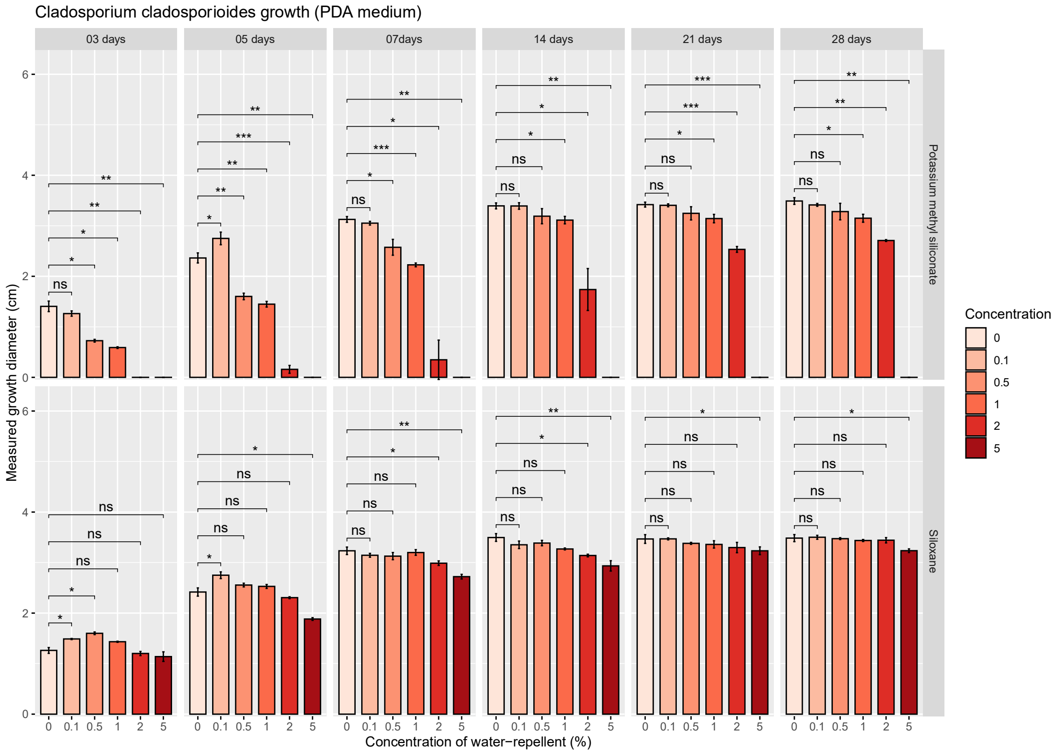

Figure S2. Monitoring of fungal growth (*Cladosporium cladosporioides*) on PDA after 3, 5, 7, 14, 21, and 28 days with increasing concentrations of PMS and Sx (0, 0.1, 0.5, 1, 2, and 5%).

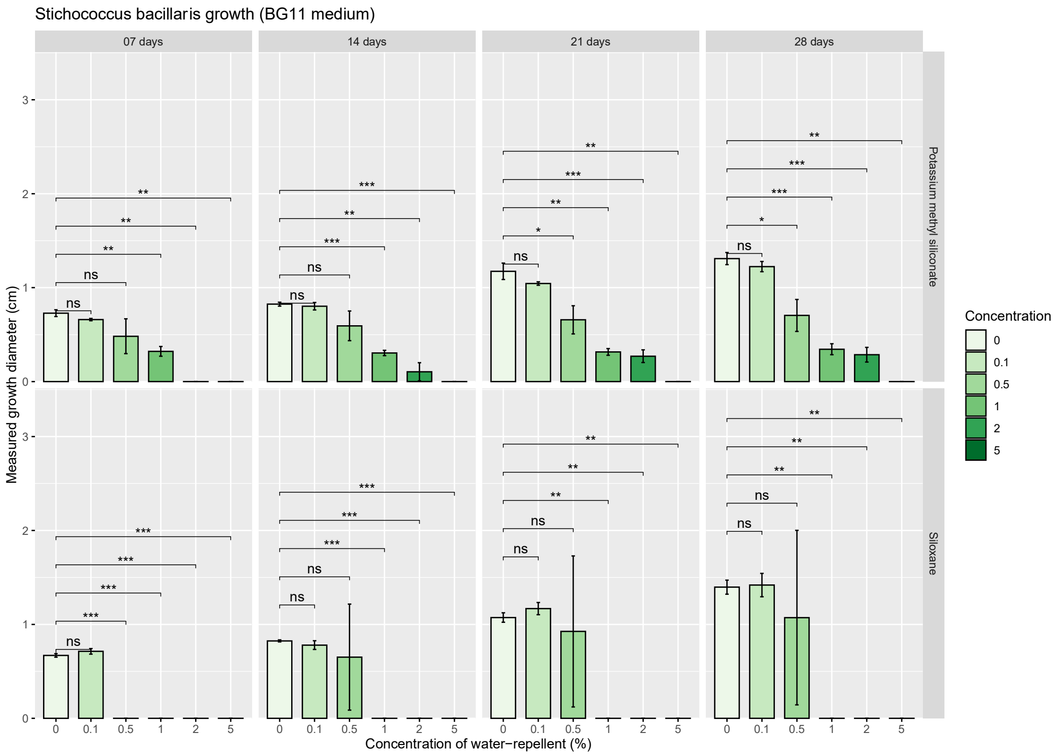

Figure S3. Monitoring of algal growth (*Stichococcus bacillaris*) on BG11 after 7, 14, 21, and 28 days with increasing concentrations of PMS and Sx (0, 0.1, 0.5, 1, 2, and 5%).

Table S1. EDS analysis of relevant spectra on UT, PMS, and Sx tiles after 6 years of exposure in Occitanie (atomic %).

	**UT**		**PMS**		**Sx**	
Spectrum Label	Tile	Colonization	Tile	Colonization	Tile	Colonization
C	8.77	31.03	6.03	27.73	6.03	19.52
O	63.10	66.33	62.74	64.09	62.67	62.27
Na	0.35	0.07	0.47	1.39	0.48	0.59
Mg	1.28	0.13	1.16	0.49	1.21	1.11
Al	8.04	0.38	7.77	1.10	8.37	3.31
Si	14.19	1.64	16.37	2.07	16.23	6.58
P	0.11	0.01	0.19	0.10	0.09	0.19
S	0.01	0.01	0.00	0.24	0.01	0.23
Cl	0.10	0.15	0.06	2.00	0.20	3.33
K	1.71	0.12	2.40	0.32	2.02	0.94
Ca	0.30	0.03	0.39	0.08	0.39	0.47
Ti	0.16	0.01	0.41	0.04	0.21	0.13
Fe	1.87	0.11	2.03	0.35	2.10	1.32
Total (% atomic)	100.00	100.00	100.00	100.00	100.00	100.00
